# Evaluation of the Antioxidant and Antiglycation Effects of *Lactarius deterrimus* and *Castanea sativa* Extracts on Hepatorenal Injury in Streptozotocin-Induced Diabetic Rats

**DOI:** 10.3389/fphar.2017.00793

**Published:** 2017-10-31

**Authors:** Jelena Arambašić Jovanović, Mirjana Mihailović, Aleksandra S. Uskoković, Nevena Grdović, Svetlana Dinić, Goran Poznanović, Ibrahim Mujić, Melita Vidaković

**Affiliations:** ^1^Department of Molecular Biology, Institute for Biological Research, University of Belgrade, Belgrade, Serbia; ^2^Department of Agriculture, University of Rijeka, Rijeka, Croatia

**Keywords:** diabetes, *Lactarius deterrimus*, *Castanea sativa*, hepatorenal protection, antioxidant and antiglycating activity

## Abstract

The present study aimed to investigate the beneficial effects of the treatment with extracts from the edible mushroom *Lactarius deterrimus* (Ld) and the chestnut *Castanea sativa* (Cs), separately and in combination (MIX Ld/Cs), on oxidative stress and advanced glycation end-product (AGE)-mediated hepatorenal injury in a rat model of streptozotocin (STZ)-induced diabetes by examining pathways responsible for maintenance of redox homeostasis. An experimental model of diabetes was induced in rats by the administration of 40 mg/kg STZ intraperitoneally (i.p.) for 5 consecutive days. The examined extracts were applied separately at a dose of 60 mg/kg i.p. and in combination (60 mg/kg each extract; i.p.) for 4 weeks, starting from the last day of STZ administration. The improvement of hepatorenal function in diabetic rats treated with the extracts was associated with an improved glycemic and lipid status and suppression of oxidative stress and thereby oxidative damage of lipids and DNA. Besides the fact that both extracts inhibited protein glycation and AGE formation *in vitro*, they also reduced non-enzymatic glycosylation in diabetic rats *in vivo.* The observed antiglycation activity of the examined extracts (separately and in combination) was accompanied with the inhibition of CML-mediated RAGE/NF-κB activation and reduction of enzymatic *O*-GlcNAcylation in liver and kidney tissues of diabetic rats. Taken together, these results reveal that the administration of chestnut and mushroom extracts, either individually or together, activates a coordinated cytoprotective response against diabetes-induced hepatorenal injury not only through recovery of the antioxidant defense system of the cell, but also through a marked antiglycation activity.

## Introduction

Diabetes mellitus (DM) type 1 is a metabolic disorder characterized by a severe deficiency in insulin secretion resulting from atrophy of the islets of Langerhans and causing hyperglycemia that results from a disruption of insulin-signaling because of insufficient insulin secretion. In many tissues, increased glucose levels enhance oxidant production that through interactions with non-enzymatic, enzymatic and mitochondrial pathways can overwhelm cellular antioxidant defenses, leading to chronic oxidative stress (King and Loeken, 2004). Perturbed insulin-signaling, elevated levels of blood glucose and increased oxidant levels arising from different sources, stimulate pathways that under physiological conditions are either not expressed or are expressed at considerably lower levels. These include stimulation of polyol pathway, increased formation of AGEs (advanced glycation end-products), increased expression of the receptor for AGEs (RAGE) and its activating ligands, activation of protein kinase C and overactivity of the hexosamine biosynthetic pathway (HBP) (Mohora et al., 2007). These different pathways are interrelated and in a pathological setting potentiate each other, leading to excess production of compounds that cause cellular damage by oxidizing membrane lipids, modifying intracellular proteins and their functions and inducing DNA damage. Ultimately, all these process are involved in the development of diabetic pathologies (Giacco and Brownlee, 2010) that results from microvascular (diabetic retinopathy, nephropathy, and neuropathy) and macrovascular complications (ischemic heart disease, cerebrovascular and peripheral vascular disease). Also, diabetes is estimated to be the most common cause of oxidative stress-related liver disease (Rutter, 2000; Guven et al., 2006).

Glucose control and the selective use of antioxidants with defined modes of action suppress the activation of oxidative stress-related pathways and delay the progression of diabetes-related complications. Established antioxidants such as *N*-acetylcysteine, vitamins C and E, and α-lipoic acid have been shown to be moderately effective in reducing certain diabetic complications, which points to an overall beneficial effect of antioxidant intake in diabetes (da Silva et al., 2010; Arambasic et al., 2013; Dinic et al., 2013). Several reports have shown that as might logically be anticipated, combinations of different antioxidants are superior to monotherapy (Kowluru and Kennedy, 2001). For that account many plant extracts and plant products have been viewed as promising therapeutic drug in the prevention of different pathologies linked to oxidative stress (Saeed et al., 2012). This is because the levels of phenolic and flavonoid compounds, highly present in plant extracts, correlate positively with its antioxidant capacity which manifests as free radical scavenging, metal ion chelation, hydrogen donation and reducing activities (Diaz et al., 2012). Several studies have also demonstrated that the phenolic content of plant extracts correlates with its anti-glycation activity (Harris et al., 2011), and that a wide range of other non-phenolic compounds such as terpenes, carotenoids, polyunsaturated fatty acids, and polysaccharides are capable of reducing non-enzymatic protein glycosylation (Sun et al., 2010). As purified bioavailable phenolic and flavonoid compounds are difficult to obtain, and because extracts have better antioxidant activities than their individual components (Calliste et al., 2005), there is a growing interest for assessing the effects and considering the potential use of plant extracts containing complex mixtures of different bioactive molecules.

Sweet chestnut tree from the Fagaceae family, which grows in the Mediterranean region of Europe, is a known source of phenolic bioactive compounds, in particular of tannins (Sanz et al., 2010). It has been widely used in folk medicine for treating various respiratory diseases and recent studies have shown that the *Castanea sativa* extract possesses antiviral (Lupini et al., 2009) and antioxidant effect (Frankič and Salobir, 2011; Mujić et al., 2011) as well as the ability to prevent DNA damage (Grdović et al., 2012). Edible and medicinal mushrooms have various biological activities and for centuries have been used in prevention and treatment of various diseases (Lindequist et al., 2005). Edible mushrooms and their constitutive active compounds have been described to have antioxidant properties and therefore are important in the management of diabetes (Yamac et al., 2008; Lo and Wasser, 2011). *Lactarius deterrimus*, also known as false saffron milkcap or orange milkcap, from the family Russulaceae is distributed in Europe, but has also found in parts of Asia. Lactarius species have been reported to exhibit a variety of biological activities including antitumor, antioxidant, and immunostimulant activity (Athanasakis et al., 2013; Hou et al., 2013).

In our previous works, we described the antioxidant properties and beneficial effects of extracts obtained from the edible mushroom *Lactarius deterrimus* (Ld), the spiny burrs of the sweet chestnut *Castanea sativa* (Cs) and their combination (MIX Ld/Cs), on streptozotocin (STZ)-induced rat pancreatic β-cell death *in vitro* (Mujić et al., 2011; Grdović et al., 2012). We observed that the strong antioxidant effect of the Cs extract corresponded to the high content of phenolic and flavonoid compounds, while the Ld extract with a low phenolic and flavonoid content had only a moderate antioxidant effect. However, the combination of these extracts (MIX Ld/Cs) significantly increased β-cell viability *in vitro* after the STZ treatment as a result of the significant reduction of DNA damage and improved redox status. We concluded that improved cytoprotection provided by MIX Ld/Cs was the consequence of additive and synergistic effects of the different antioxidant activities, contained in the chestnut and mushroom extracts. To lend credence to the potential *in vivo* beneficial biological effects of the mushroom and chestnut extracts, we investigated the effect of their daily administration for 4 weeks, either separately (Cs or Ld) or combined (MIX Ld/Cs), on the pathways responsible for redox homeostasis maintenance in the liver and kidney of STZ-induced diabetic rats.

## Materials and Methods

### Chestnut and Mushroom Material and Extraction Procedures

The mushroom *Lactarius deterrimus* (Ld) was collected in the Istra region in Croatia, in the summer of 2008. Fruiting bodies were gently cleansed of any residual compost, air-dried and stored in airtight plastic bags at room temperature. Samples of spiny burrs of the sweet chestnut (Cs) (*C. sativa* Mill.) were collected in western Bosnia and Herzegovina. The chestnut samples were collected during the chestnut-ripening season, from the middle of September to the end of October 2006. The collected samples were kept at -20°C and protected from light before further use. The dried mushroom samples and chestnut samples (spiny-burrs) was obtained from Dr. Senka Vidović (Faculty of Technology, University of Novi Sad) The dried samples were milled in a blender before extraction with 50% ethanol, at a sample:solvent ratio of 1:10 (w/v) for the mushroom extract, and 1:5 (w/v) for the chestnut extract. The extraction process was carried out using an ultrasonic bath (B-220; Branson and SmithKline Company) at 45°C for 40 min for the mushroom extract, and at room temperature for 30 min for the chestnut extract. After filtration, the extraction solvent was removed using a rotary evaporator (Devarot; Elektromedicina) under vacuum. The obtained extracts were dried at 60°C to a constant mass and stored in glass bottles at -80°C to prevent oxidative damage.

### Phytochemical Analysis of *Castanea sativa* and *Lactarius deterrimus* Extracts

The contents of total phenolic compounds in the dry mushroom and chestnut extracts were determined by the Folin–Ciocalteu procedure at 765 nm (Singleton and Rossi, 1965). The values are expressed as mg of gallic acid equivalents (GAE) per 1 g of dry extract. The Cs and Ld extracts contained 252 and 14.8 mg gallic acid per gram of dry material, respectively, in total phenolics. The total flavonoid contents were established by the aluminum-chloride colorimetric assay at 510 nm (Markham, 1989). The values are expressed as mg of quercetin equivalents (QE) per 1 g of dry extract. The flavonoid contents in the Cs and Ld extracts were 192 and 5.07 mg quercetin per gram of dry material, respectively. Previous detailed qualitative and quantitative analyses of the extract by High-Performance Liquid Chromatography with Diode-Array Detection (HPLC/DAD) revealed in Cs extract the highest content of ellagic acid structures, followed by gallic acid derivatives and flavonoid structures (Grdović et al., 2012). In the Ld extract, compounds containing a phenolic group were tryptophan and *p*-hydroxybenzoic acid, while other detected compounds were unsaturated and oxy (hydroxy- or epoxy-) fatty acids (Grdović et al., 2012).

### Experimental Protocol

Experiments were performed on 2.5-month-old adult albino Wistar rats weighing 220–250 g. All animal procedures were approved by the Committee for Ethical Animal Care and Use of the Institute for Biological Research “Siniša Stanković,” University of Belgrade, which acts in accordance with the EEC Directive (86/609/EEC) on the protection of animals used for experimental and other scientific purposes. The experimental model of multiple low-dose STZ-induced diabetes was used. Diabetes was induced by intraperitoneal (i.p.) injections of 40 mg STZ/kg to Wistar rats for 5 consecutive days. STZ was dissolved immediately before use in sodium citrate buffer (0.1 M, pH 4.5). Blood glucose was measured 24 h after the last STZ injection. Blood samples were obtained from the tail vein of overnight-fasted rats, and glucose was measured with a blood glucometer (Accu-Chek Active). Rats were considered to have diabetes when the fasting blood glucose level exceeded 20 mmol/l. Male Wistar rats were randomly divided into five groups: (i) ND – the non-diabetic control group (*n* = 7) which received an equivalent volume of citrate buffer i.p. for 5 consecutive days; (ii) D – the diabetic group (*n* = 8) that received STZ (40 mg/kg/5 days, i.p.) and was left untreated throughout the 4-week period; (iii) D + Cs – the diabetic group treated with the chestnut extract (Cs; 60 mg/kg, i.p.) daily for 4 weeks (*n* = 8), starting from the last day of STZ administration; (iv) D + Ld – the diabetic group treated with the mushroom extract (Ld; 60 mg/kg, i.p.) daily for 4 weeks (*n* = 8), starting from the last day of STZ administration; (v) D + Cs/Ld – the diabetic group treated with the combination of chestnut and mushroom extract (Mix Cs/Ld; 60 + 60 mg/kg, i.p.) daily for 4 weeks (*n* = 8), starting from the last day of STZ administration. The mode of administration was chosen to be i.p. because rats refused to drink the extract due to its bitterness. Based on our previous experimental experience, we estimated that i.p. administration is less stressful for the rats than force-feeding by gavage and it ensures that animal receive the desired dose. Even dough, the i.p. delivery is considered a parenteral route of administration, the pharmacokinetics of substances administered intraperitoneally are more similar to those seen after oral administration (Turner et al., 2011) as well as the bioavailability of substances is higher. The rats were killed 4 weeks after diabetes was established. Following blood collection, an abdominal incision was made and the liver and both kidney were removed and processed further as indicated. The remaining liver and kidney tissue was frozen in liquid nitrogen, kept at -80°C and used for cytoplasmic and nuclear protein preparation according to the instructions described in ProteoJET cytoplasmic and nuclear protein extraction kit (Fermentas).

### Determination of Biochemical Parameters of Diabetes and Histological Analysis of Liver and Kidney Tissue

After 4 weeks of diabetes, the rats were fasted overnight and their blood was collected in heparinized tubes (1,000 IU heparin). Blood plasma was obtained after centrifugation at 2,000 × *g* for 10 min and used for determination of glucose, total cholesterol, triglycerides, aspartate and alanine aminotransferases (AST and ALT, respectively), and blood urea nitrogen (BUN). After centrifugation and collection of plasma fractions, the remaining pellets were used for the preparation of red blood cell (RBC) hemolysates. RBC were washed twice with 0.9% NaCl and lysed in three volumes of cold water for 30 min on ice. Hemolysates were used for the determination of glycated (Gly) hemoglobin (Hb).

Blood glucose concentrations were measured with a commercial kit (Gluco-quant Glucose/HK, Boehringer Mannheim, Germany), which is based on the hexokinase/G6P-DH enzymatic method. Concentrations of GlyHb were determined by the colorimetric assay according to Parker et al. (1981). Serum triglyceride concentrations were determined by the glycerol-3-phosphate (GPO) oxidase-*p*-aminiphenazone (PAP) method with an enzymatic kit (Randox Laboratories, United Kingdom). Total cholesterol concentrations were measured by the enzymatic cholesterol esterase-cholesterol oxidase method using the CHOD-PAP test kit (Randox Laboratories, Crumlin, United Kingdom). Activities of ALT and AST were estimated by measuring the produced oxaloacetate and pyruvate, respectively, using an optimized standard UV kinetic method kit [GPT (ALAT) IFCC mod.; GOT (ASAT) IFCC mod.]. Blood urea nitrogen level was estimated according to GLDH method (Human, Wiesbaden, Germany).

The liver and kidney tissue from all experimental groups were removed and fixed in 10% buffered formalin for histological examination. Liver and kidney tissues from all examined groups were embedded in paraffin wax and sectioned at 5 mm thickness for histological examination. For histological analysis tissue sections were stained with Hematoxylin (H) and Eosin (E), mounted and observed under a light microscope (Leica DMLB; objective magnification 20×).

### Estimation of Oxidative Stress

The level of oxidative stress in liver and kidney tissue was estimated by using 2′,7′-dichlorofluorescin diacetate (DCFDA), a fluorogenic dye that measures hydroxyl, peroxyl, and other reactive oxygen species (ROS) activity. Briefly, an aliquot of the liver and kidney homogenates (0.01 ml) were mixed with 0.15 ml ethanol solution of DCFDA to the final concentration of 10 mM. After incubation for 30 min at RT and in dark the fluorescence was measured using Tecan microplate reader Safire II (excitation and emission wavelengths of 488 and 520 nm, respectively).

### Thiobarbituric Acid-Reactive Substance (TBARS) Assay

Lipid peroxidation was examined with the TBARS assay according to Ohkawa et al. (1979). Briefly, an aliquot of the liver and kidney homogenates (0.1 ml) prepared in 1.15 M KCl were mixed with 0.2 ml of 8.1% SDS, 1.5 ml of 20% acetic acid (pH 3.5), 1.5 ml of 0.8% TBA, and 0.7 ml water and heated at 95°C for 60 min. After cooling to room temperature, 1 ml of water and 5 ml of *n*-butanol/pyridine (15:1, v/v) were added, mixed and centrifuged at 3,000 × *g* for 10 min. The red pigment in the supernatant fractions was estimated by absorbance at 532 nm. A calibration curve was prepared with 1,1,3,3-tetramethoxypropan (concentrations ranged from 25 nmol/ml to 1 μmol/ml).

### Comet Assay

The level of DNA damage was assessed by the alkaline Comet assay (Singh et al., 1988). Liver and kidney samples from all experimental groups were minced in ice-cold HBSS buffer (0.14 g/l CaCl_2_, 0.4 g/l KCl, 0.06 g/l KH_2_PO_4_, 0.1 g/l MgCl_2_ × 6H_2_O, 0.1 g/l MgSO_4_ × 7H_2_O, 8.0 g/l NaCl, 0.35 g/l NaHCO_3_, 0.09 g/l Na_2_HPO_4_ × 7H_2_O, 1.0 g/l D-glucose) containing 20 mM EDTA and 10% DMSO. Ten microliters of a liver or kidney cell suspension was mixed with low-melting agarose (0.75%) and applied to a microscope slide. Cells were lysed for 2 h at 4°C in lysis buffer (2.5 M NaCl, 100 mM EDTA, 10 mM Tris, pH 10, 1% Triton X-100). After lysis, the slides were incubated for 30 min at 4°C in electrophoresis solution (300 mM NaOH, 1 mM EDTA, pH 13.0) and subjected to electrophoresis in order to separate the damaged DNA fragments. The slides were placed in neutralization buffer (0.4 M Tris-HCl, pH 7.4) and stained with SYBR Green I (1:10,000 dilution; Sigma–Aldrich). Fluorescence microscopy was performed with a Zeiss Axiovert 135TV microscope equipped for epifluorescence. DNA damage was quantified by measuring the displacement between the genetic material contained inside the nuclear sphere or comet ‘head’ and the resulting comet ‘tail.’ Images were analyzed with TriTekCometScore Freeware v1.5.^1^

### Formation of Fructosamines during *in Vitro* Glycation

Reaction mixtures containing bovine serum albumin (BSA) (20 mg/ml)/glucose (1 M)/fructose (1 M) system (1 ml), dissolved in 20 mM Tris-HCl (pH 7.4), in the presence or absence of examined extracts were incubated at 37°C for 7 days (Brownlee et al., 1986). The incubated solution was mixed with 0.5 mM NBT (0.5 ml) dissolved in 100 mM carbonate puffer pH 10.8. The reduction of NBT was measured at 595 nm according to the formula: %Inhibition = ((*A*_blank_ - *A*_test_)/*A*_blank_) × 100, where *A*_blank_ is the absorbance of the BSA/glucose system without the test sample, and *A*_test_ is the absorbance of BSA/glucose system in the solution with the test sample.

### AGE Formation *in Vitro*

Bovine serum albumin (20 mg/ml) was incubated with glucose (1 M) and fructose (1 M) in Tris-HCl pH 7.4 containing 0.02% sodium azide. All of the reagents and the examined extracts were passed through filter paper and each of the mixtures was incubated at 37°C for 7 days. The formation of AGE was measured by the fluorescence intensity at an excitation wavelength of 330 nm and an emission wave-length of 410 nm with a Luminescence spectrometer LS50B (PerkinElmer Ltd., Buckinghamshire, England) (Lee et al., 1998).

### Determination of Superoxide Dismutase (SOD) and Catalase (CAT) Activities

For determination of antioxidant enzyme activities, a homogenate of fresh liver and renal tissue was prepared in sucrose buffer (0.25 M sucrose, 1 mM EDTA, 0.05 M Tris-HCl, pH 7.4). Firstly, 1 g of liver and kidney tissue was homogenized in 10 ml of sucrose buffer, sonicated, and at the end centrifuged at 100,000 × *g* in a Beckmann rotor Ti 50 for 90 min. Total SOD activity was measured by the epinephrine method (Misra and Fridovich, 1972). SOD activity was expressed as U/mg of protein. MnSOD activity was performed after preincubation with 8 mmol/l KCN. CuZnSOD activity was calculated from the difference between total SOD and MnSOD activities. CAT activity was measured by the rate of hydrogen peroxide decomposition and expressed as l M H_2_O_2_/min/mg protein (U/mg of protein) (Claiborne, 1984). Protein concentrations were determined according to Lowry et al. (1951).

### Determination of Reduced to Oxidized Glutathione Ratio (GSH/GSSG)

Total GSH and GSSG levels were determined by the standard recycling method using the Glutathione Assay Kit (Cayman Chemicals Company, Ann Arbor, MI, United States). A 20% homogenate of fresh liver and renal tissue was prepared in phosphate buffer (100 mM NaH_2_PO_4_, 1 mM EDTA, pH 7.5). Samples for GSSG determination were incubated at room temperature with 2 μl of 0.5 M 2-vinyl pyridine solution per 50 μl of deproteinized sample for 1 h. Samples were deproteinized with 5% 5-Sulfosalicylic acid solution. Incubation with 2-vinyl pyridine conjugates any GSH present in the sample so that only GSSG is recycled to GSH. The GSSG was then subtracted from the total GSH to determine the actual GSH level and GSH/GSSG ratio.

### Sodium Dodecyl Sulfate (SDS)–Polyacrylamide Gel Electrophoresis (PAGE) and Immunoblot Analysis

For immunoblot analysis, homogenates of fresh liver and renal tissue prepared in sucrose buffer were used. Twenty micrograms of liver and kidney protein fractions were separated onto 4% stacking/12% separating slab gels. After electrophoresis, proteins were transferred to PVDF membranes (Hybond-P, Amersham Pharmacia Biotech) and immunoblot analysis was performed using rabbit polyclonal antibodies to *O*-linked β-*N*-acetylglucosamine (*O*-GlcNAc), *N*-(carboxymethyl)lysine (CML), RAGE, nuclear factor kappa-light-chain-enhancer of activated B cells (NF-κB) p65, β-actin; goat polyclonal anti-bodies to lamin B (Santa Cruz Biotechnology, Santa Cruz, CA, United States). Appropriate horseradish peroxidase-conjugated anti-rabbit, anti-goat (Santa Cruz Biotechnology, Santa Cruz, CA, United States), and anti-mouse (Cell Signaling) immunoglobulins were used as a secondary antibody. Each PVDF membrane was reprobed according to the supplier’s protocol for reprobing. Immunoreactive bands were identified by an enhanced chemiluminescence (ECL) detection system (Santa Cruz Biotechnology) according to the manufacturer’s instructions. The blots were scanned, and the intensities of the signals were quantified using TotalLab (Phoretix, Newcastle Upon Tyne, England) electrophoresis software (ver. 1.10).

### Statistical Analysis

The data were expressed as the mean ± SEM (standard error of mean). Before statistical analysis we tested the obtained data for normality using a Kolmogorov–Smirnov test. For intergroup comparison between two means, first a one-way Analysis of Variance (ANOVA) was used and in case of significance a Duncan’s multiple range test (DMRT) was applied as a *post hoc* test. The difference was considered statistically significant at *p* < 0.05. Mann–Whitney test was used in one particular case (for total amount of Nf-kB) to compare differences between two independent groups (*p* < 0.05).

## Results

### The Effects of Extracts on the Biochemical Parameters and Histological Features of Liver and Kidney Tissue in Diabetic Rats

The biochemical markers of diabetes in different experimental groups are presented in **Table 1**. Compared to the control non-diabetic rats, the STZ-treated animals displayed typical signs of diabetes: elevated fasting blood glucose and total glycated hemoglobin level (5- and 2-fold, respectively), as well as increased total cholesterol and triglyceride concentrations (1.6- and 1.9-fold, respectively). The activation of detrimental processes in the liver and kidney of diabetic rats was observed as increased enzymatic activities of ALT and AST, markers of hepatocellular injury, and increased BUN, an indicator of renal functionality. The treatment of diabetic rats with Cs extract lowered fasting blood glucose, GlyHb, cholesterol, triglyceride and ALT levels to the control values and significantly lowered the concentration of BUN. In contrast, the treatment of diabetic rats with the Cs extract did not cause any significant change in AST activity. Administration of the Ld extract to diabetic rats alleviated hyperglycemia, decreased GlyHb and triglyceride levels and restored the total cholesterol concentration to the control values. While the treatment of diabetic rats with the Ld extract improved the enzymatic activity of AST, the level of ALT activity and BUN concentration remained elevated. Diabetic rats treated with the combination of the mushroom and chestnut extracts MIX Ld/Cs displayed improvements in all measured parameters, indicating a better glycemic and lipid status and improved liver and kidney functioning.

**Table 1:** Effect of *Castanea sativa, Lactarius deterrimus*, and their combination on biochemical parameters in streptozotocin (STZ)-induced diabetic rats.

	ND	D	D + Cs	D + Ld	D + Cs/Ld
Glucose (mmol/L)	6.2 ± 0.22^a^	30.1 ± 1.12^b^	6.2 ± 0.25^a^	22.7 ± 0.94^c^	19.6 ± 0.91^c^
GlyHb (mmol/gHb)	6.5 ± 0.26^a^	11.6 ± 0.51^b^	7.7 ± 0.32^a^	9.1 ± 0.41^c^	8.7 ± 0.38^c^
Cholesterol (mmol/L)	1.6 ± 0.07^a^	2.6 ± 0.11^b^	1.7 ± 0.05^a^	1.6 ± 0.04^a^	1.9 ± 0.09^c^
Triglycerides (mmol/L)	0.7 ± 0.03^a^	1.4 ± 0.06^b^	0.7 ± 0.02^a^	1.0 ± 0.05^c^	1.1 ± 0.04^c^
AST (U/L)	199.1 ± 4.5^a^	284.1 ± 7.2^b^	267.4 ± 5.5^b^	250.4 ± 6.3^c^	255.3 ± 5.2^c^
ALT (U/L)	58.6 ± 1.6^a^	113.3 ± 3.4^b^	59.4 ± 1.5^a^	116.4 ± 3.5^b^	90 ± 2.9^c^
BUN (mmol/L)	6.8 ± 0.26^a^	14.6 ± 0.65^b^	12.3 ± 0.52^c^	14.35 ± 0.6^b^	11.9 ± 0.45^c^


*ND, non-diabetic rats; D, diabetic rats; Cs, *Castanea Sativa*; Ld, *Lactarius deterrimus.**

*Values are means ± SEM; values not sharing a common superscript letter differ significantly at *p* < 0.05 (Duncan’s multiple range test, DMRT).*

**FIGURE 1:**
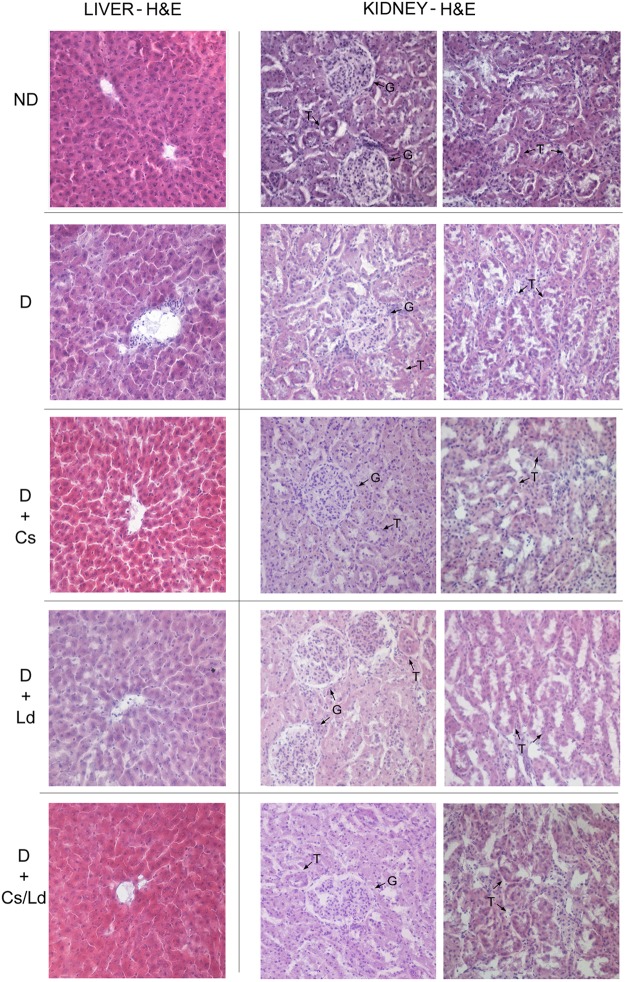
The effect of *Castanea sativa, Lactarius deterrimus* extracts and their combination on the histological changes in liver and kidney tissue of diabetic rats. H&E-hematoxylin and eosin staining of liver and kidney sections (magnification 20×); ND, non-diabetic control rats; D, diabetic rats; D + Cs, diabetic rats treated with *C. sativa*; D + Ld, diabetic rats treated with *L. deterrimus*; D + Cs/Ld, diabetic rats treated with the combination of *C. sativa* and *L. deterrimus* extract; G, glomerulus; T, tubules.

**Figure 1** depicts the photomicrographs of hematoxylin and eosin staining of liver and kidney tissues of control and experimental groups of rats. ND control rats showed liver parenchyma with general structures preserved, including hepatic lobules with normal hepatocytes surrounded by sinusoids and distributed radially toward the centrilobular veins with no observed inflammatory infiltration and fatty degeneration. In contrast, untreated D rats presented morphological changes in the liver that were characterized by hepatocytes that contained focal or generalized fatty vacuoles and micro- or macro vesicular features that were associated with the presence of dilated sinusoids and a progressive loss of general organ structure (disorganization of the lobular architecture) together with mild inflammatory cell infiltration. Histological examination of the livers of diabetic rats treated with Cs extract and MixCs/Ld maintained normal lobular architecture and had mild fatty change, feathery degeneration, and only slight neutrophil infiltration almost comparable to the normal. Also, Ld treatment protected liver tissue from diabetes-induced damage, but to a lesser extent.

The sections of kidney tissue of control rats demonstrated normal architecture with normal glomeruli and tubules. On the other hand, section of kidney tissues of diabetic group of rats revealed visible distortion in the architecture of the kidney tissue showing features of glomerulopathy such as compression of the glomerular tuft into nodules with decreased cellularity of the glomeruli, dilated tubules lined by cuboidal epithelium without brush borders and marked inflammatory cells infiltration. However, the treatment of diabetic rats with Cs extract and MixCs/Ld attenuated the histological changes in the kidney of diabetic rats showing better patterned renal architecture with fairly normal glomeruli and tubules and mild inflammatory cells infiltration. Also, Ld treatment attenuated kidney damage, but to a lesser extent.

### The Beneficial Effects of Extracts on Hyperglycemia-Induced Oxidative Stress in the Liver and Kidney of Diabetic Rats

**FIGURE 2:**
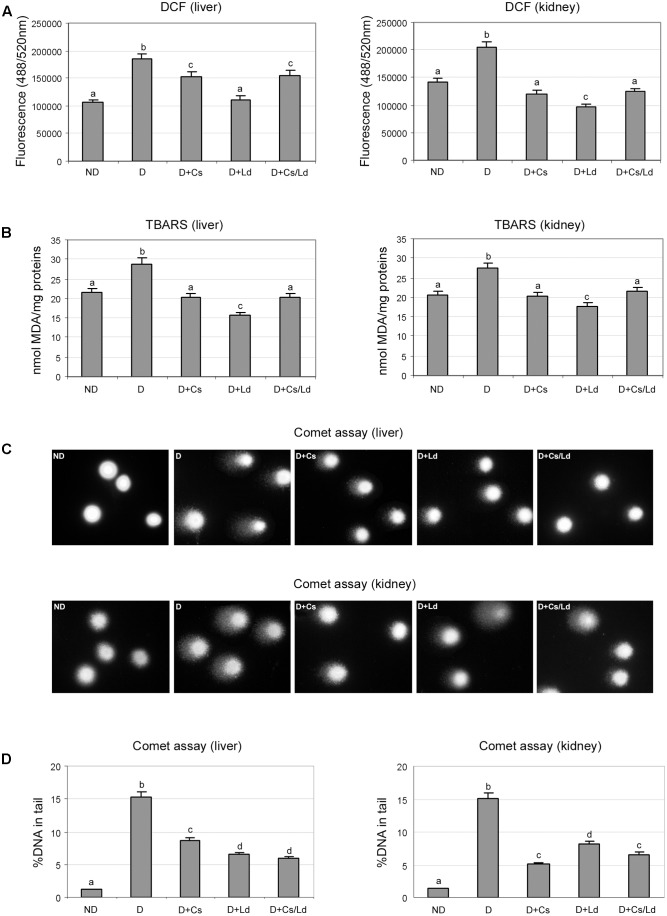
The effect of *C. sativa, L. deterrimus* extracts and their combination on the level of oxidative stress and oxidative damage of lipids and DNA in liver and kidney tissues. **(A)** Oxidative stress was estimated by 2′,7′-dichlorofluorescin diacetate (DCFDA). **(B)** Lipid peroxidation was estimated by the thiobarbituric acid-reactive substance (TBARS) assay. **(C)** Representative images of DNA damage assessed by the alkaline Comet assay. **(D)** Assessment of DNA damage using % of DNA in comet’s tail as a parameter of DNA damage. ND, non-diabetic control rats; D, diabetic rats; D + Cs, diabetic rats treated with *C. sativa*; D + Ld, diabetic rats treated with *Lactarius deterrimus*; D + Cs/Ld, diabetic rats treated with the combination of *C. sativa* and *L. deterrimus* extract. Results are expressed as mean ± SEM. Means not sharing a common letter are significantly different between groups (Duncan’s multiple range test, DMRT; *p* < 0.05).

To estimate the level of oxidative stress we used a non-fluorescent substrate 2′,7′-dichlorofluorescein (DCFDA) which undergoes oxidation to green fluorescent product in the presence of ROS. The highest level of oxidative stress, based on the measured fluorescence, was detected in the liver and kidney of diabetic animals (**Figure 2A**). On the other hand, in the liver and kidney tissue of diabetic animals treated with the extracts, separately and in combination, the extent of ROS production was significantly lower. The strongest effect on the ROS production in both examined tissues was displayed by mushroom extract (**Figure 2A**).

The end products of lipid peroxidation are reactive aldehydes, such as MDA, which served as an indicator of lipid peroxidation in liver and kidney in each experimental group (**Figure 2B**). Highest concentration of MDA equivalents (MDAeq), measured using the thiobarbituric acid-reactive substance assay, were detected in the liver and kidney of diabetic rats. Comparison of MDAeq levels in the liver and kidney of diabetic animals and the diabetic animals treated with the extracts revealed that both extracts, as well as MIX Ld/Cs, exhibited statistically significant inhibition of lipid peroxidation. The mushroom extract showed the highest potential for inhibition of lipid peroxidation, based on the MDAeq level that was 2-fold lower than in the liver of diabetic rats and 1.6-fold lower than in the kidney of diabetic rats. The chestnut extract and the combination of extracts were less effective, as they both induced a 1.4-fold decrease of MDAeq in both liver and kidney when compared to diabetic rats (**Figure 2B**).

Oxidative damage to DNA in the liver and kidney in each experimental group was evaluated by the alkaline Comet assay (**Figures 2C,D**). The extent of DNA damage was expressed as % of DNA in comet’s tail (**Figure 2D**). Representative images of liver and kidney cells whose DNA was visualized by staining with SYBR Green I are shown in **Figure 2C**. In the control, non-diabetic group, the majority of analyzed liver and kidney cells did not exhibit DNA damage (**Figure 2C**, ND panels). In STZ-induced diabetic rats, extensive DNA damage was observed in liver and kidney cells. This was revealed by the large amount of DNA in the comet tails (**Figures 2C,D** panels). Treatments of diabetic animals with the extracts, separately and in combination, led to a pronounced decrease in DNA damage in liver and kidney cells (panels D + Cs, D + Ld, and D + Cs/Ld). Analysis of % of DNA in comet’s tail revealed that the mushroom extract and the combination of the extracts provided the best protection against diabetes-induced DNA damage in liver cells (**Figure 2D**). On the other hand, in kidney cells, the chestnut extract and the combination of the extracts caused the greatest decrease in DNA damage (**Figure 2D**). Taken together, these results indicate that both extracts and their combination significantly suppressed oxidative stress and thereby oxidative damage of lipids and DNA in liver and kidney tissue of diabetic animals.

**FIGURE 3:**
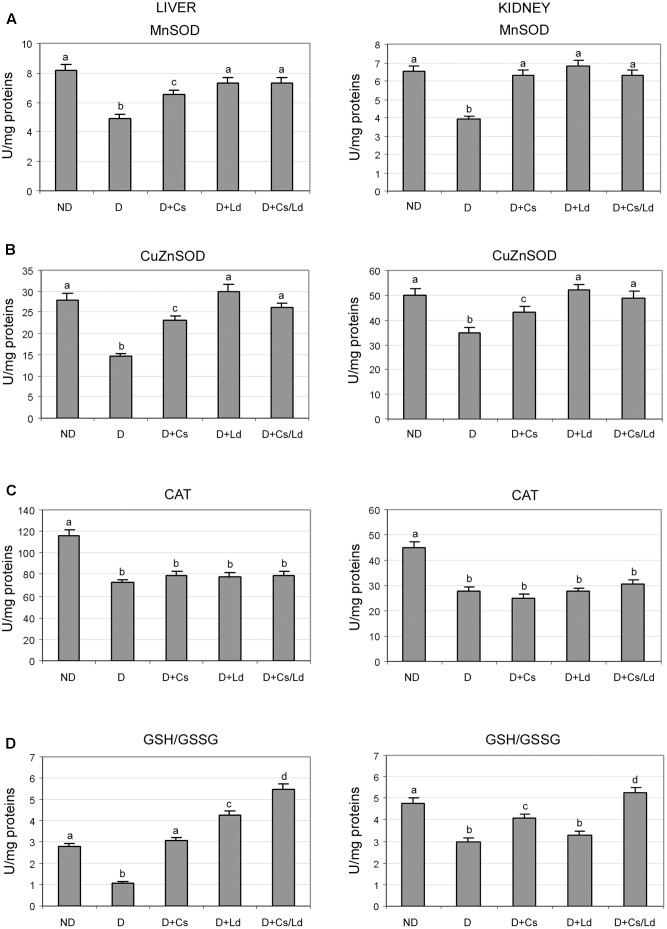
The effect of *C. sativa, L. deterrimus* extracts and their combination on the activities of antioxidant enzymes MnSOD **(A)**, CuZnSOD **(B)**, catalase (CAT) **(C)** and reduced to oxidized glutathione ratio (GSH/GSSG) **(D)** in liver and kidney tissue. ND, non-diabetic control rats; D, diabetic rats; D + Cs, diabetic rats treated with *C. sativa*; D + Ld, diabetic rats treated with *L. deterrimus*; D + Cs/Ld, diabetic rats treated with the combination of *C. sativa* and *L. deterrimus* extract. Results are expressed as means ± SEM. Means not sharing a common letter are significantly different between groups (DMRT; *p* < 0.05).

To determine whether the observed suppression of oxidative stress after the treatment with extracts was caused by changes in activities of antioxidant enzymes, we examined the activities of MnSOD, CuZnSOD, and CAT. As can be seen in **Figure 3**, STZ-induced diabetes was associated with significantly decreased MnSOD, CuZnSOD, and CAT activities in liver and kidney tissue. The treatment of diabetic rats with both extracts separately and in combination promoted increased activity of MnSOD to nearly control level in both examined tissues, except in the case of Cs-treated animals where the level of liver MnSOD activity remained below its control level (**Figure 3A**). Treatment of STZ-diabetic rats with the Ld extract and MIX Ld/Cs restored the activity of liver and kidney CuZnSOD to the control levels (**Figure 3B**). However, the chestnut extract caused a significant increase of CuZnSOD activity which was 18 and 14% below its control values in the liver and kidney, respectively. In contrast to the restoration of SOD activities, the treatments of diabetic rats with Cs, Ld, and MIX Ld/Cs did not produce any effect on CAT activity in the liver and kidney of diabetic rats (**Figure 3C**).

During oxidative stress, the concentration of GSH decreases and is accompanied by an increase in its oxidized form (GSSG). A significant decrease in the GSH/GSSG ratio was detected in the liver and kidney of diabetic rats as compared to non-diabetic controls (**Figure 3D**). The treatment of diabetic rats with either extract or their combination shifted the redox balance of glutathione in favor of its reduced form in both tissues, except in kidney tissue of Ld-treated rats. The strongest effect on the GSH/GSSG ratio in both tissues was displayed by MIX Ld/Cs (**Figure 3D**).

### Evaluation of the Antiglycation Properties of the Examined Extracts *in Vitro*

**FIGURE 4:**
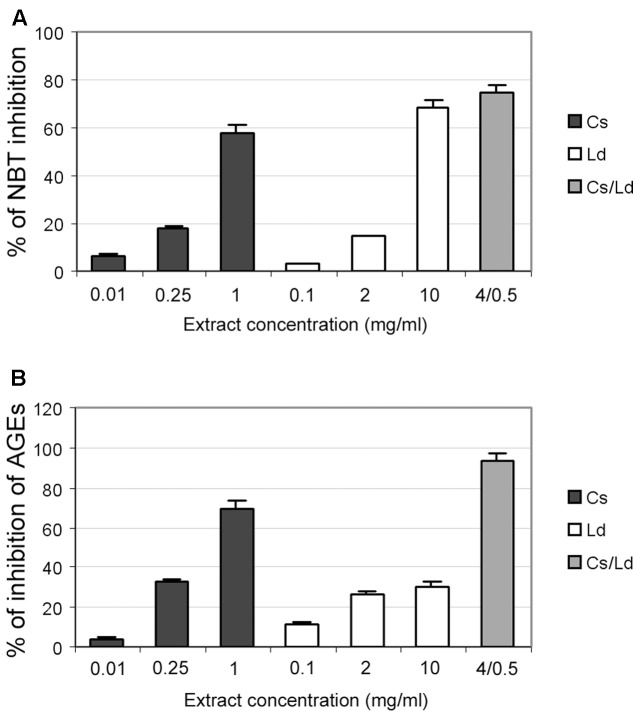
The effect of *C. sativa, L. deterrimus* extracts and their combination on non-enzymatic glycation *in vitro*. **(A)** Formation of fructosamines in the presence of different concentrations of examined extracts measured by NBT assays. **(B)** Formation of advanced glycation end-product (AGE) in the bovine serum albumin (BSA)/glucose/fructose system in the presence of different concentrations of extracts. The values are means ± SEM from three separate experiments.

We examined the potential inhibitory effects of the separate extracts and their combination on the formation of fructosamines during the process of protein glycation. This was studied by monitoring the reduction of NTB on the 7th day of incubation in the BSA/glucose/fructose system in the presence or absence of the examined extracts. The concentration ranges differed for the two extracts and were previously defined (Grdović et al., 2012). As can be seen on **Figure 4A**, the inhibition of NBT reduction, which is directly related to the ability of the examined extracts to suppress glycation, was dose-dependent in the case of both extracts, while the combination of the two extracts exhibited the strongest anti-glycation activity, providing nearly 80% inhibition of NBT reduction (**Figure 4A**). The formation of AGE was monitored by measuring the fluorescence intensity of the BSA/glucose/fructose solution attributed to the formation of glycophore groups. As shown in **Figure 4B**, when the extracts were added to the reaction media containing the BSA/glucose/fructose system, a significant concentration-dependent reduction in fluorescence intensity was observed at the end of study period. Again, the combination of different extracts displayed the greatest effect, inhibiting AGE formation by 90%.

### Effects of the Examined Extracts on Non-enzymatic Protein Glycation and AGE-Mediated Pathways in the Liver and Kidney of Diabetic Rats

**FIGURE 5:**
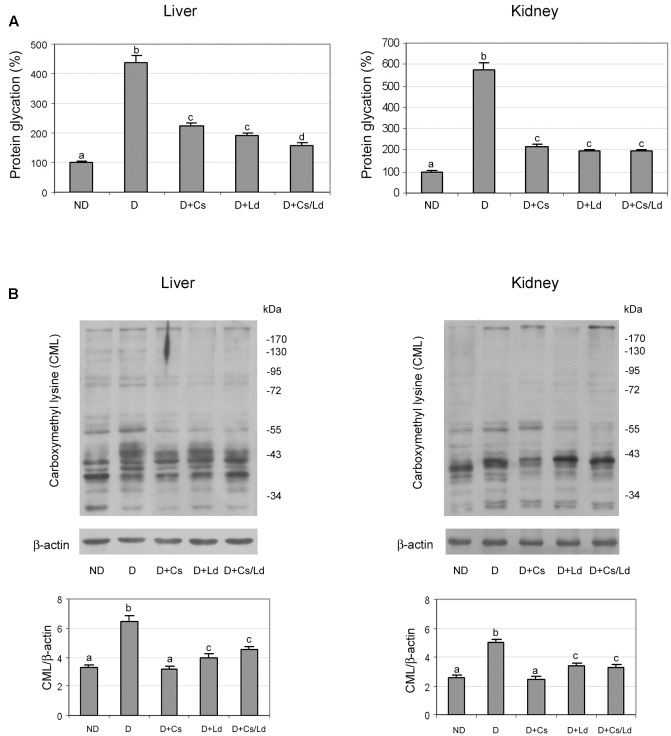
The effect of *C. sativa, L. deterrimus* extracts and their combination on protein glycation and formation *N*-(carboxymethyl)lysine (CML)-modified proteins in liver and kidney tissue. **(A)** Glycation of serum proteins measured by the fructosamine assay. **(B)** Immunoblot analysis of CML-modified proteins in whole liver and kidney homogenates. ND, non-diabetic control rats; D, diabetic rats; D + Cs, diabetic rats treated with *C. sativa*; D + Ld, diabetic rats treated with *L. deterrimus*; D + Cs/Ld, diabetic rats treated with the combination of *C. sativa* and *L. deterrimus* extracts. Results are expressed as means ± SEM. Means not sharing a common letter are significantly different between groups (DMRT; *p* < 0.05).

After establishing that both extracts inhibit protein glycation and AGE formation *in vitro*, we investigated *in vivo* protein glycation in the liver and kidney. We observed increased protein glycation in both the liver and kidney of diabetic rats in comparison to the control glycation levels (**Figure 5A**). Treatments of diabetic rats with the Cs, Ld, and MIX Ld/Cs significantly diminished the amount of glycated proteins in both examined tissue. In liver tissue, the combination of the Cs and Ld extracts provided the best protection against diabetes-induced protein glycation, while in kidney tissue all three treatments decreased glycated proteins to a similar level.

Reactive derivatives of non-enzymatic glucose-protein condensation reactions form a heterogeneous group of irreversible adducts called AGE, which refers to a broad range of advanced products of the Maillard reaction (Reinauer, 1993), including stable AGE compounds such as CML. As expected, immunostaining for CML revealed that diabetic rats exhibited significantly elevated AGE levels in the liver and kidney as compared to non-diabetic controls (**Figure 5B**). Densitometric analysis of the electrophoretic protein profiles showed that the treatment of diabetic rats with Ld, Cs, and MIX Ld/Cs reduced CML immunostaining in both tissues to significantly lower levels than in diabetic rats. The greatest reduction of CML was detected in liver and kidney tissues of diabetic animals that were treated with the Cs extract.

**FIGURE 6:**
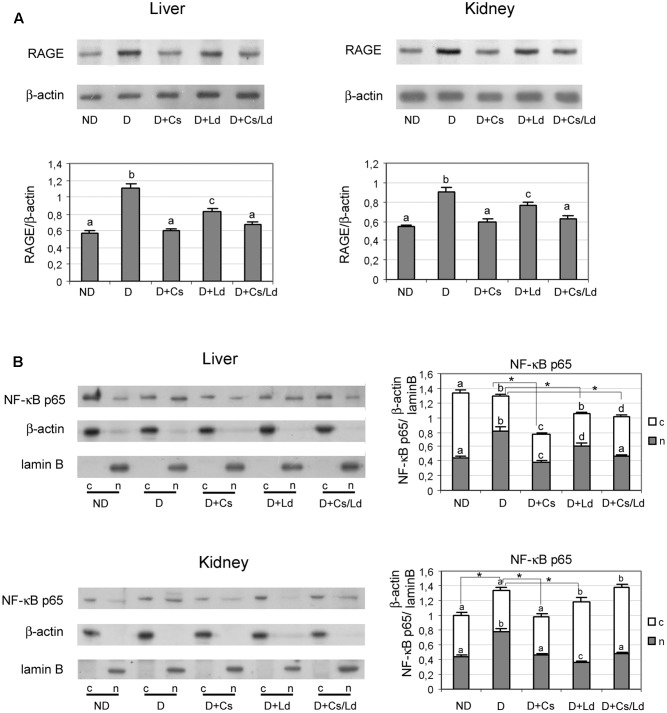
The effect of *C. sativa, L. deterrimus* extracts and their combination on the RAGE/NF-κB axis in liver and kidney tissue. **(A)** RAGE protein levels in liver and kidney homogenates and **(B)** p65 NF-κB protein levels in liver and kidney nuclear and cytosol fractions examined by immunoblot analysis. ND, non-diabetic control rats; D, diabetic rats; D + Cs, diabetic rats treated with *C. sativa*; D + Ld, diabetic rats treated with *L. deterrimus*; D + Cs/Ld – diabetic rats treated with the combination of *C. sativa* and *L. deterrimus* extract; c-cytosol; n-nucleus. Results are expressed as mean ± SEM. Means not sharing a common letter are significantly different between groups (DMRT; *p* < 0.05); ^∗^ total amount of p65 NF-κB (nuclear + cytosol) significantly different compared to diabetic rats (Mann–Whitney test, *p* < 0.05).

To examine whether the detected suppression of AGE formation after treatment of diabetic rats with extracts affected the RAGE-NF-κB axis, we performed immunoblot analysis of liver and kidney tissues with anti-RAGE and anti-NF-κB antibodies. Densitometric analysis of the bands obtained after immunoblot analysis (**Figure 6A**) shows increased levels of RAGE in liver and kidney homogenates of diabetic rats. This increase was effectively prevented by treatments of diabetic rats with the Ld and Cs extracts separately, as well as their combination MIX Ld/Cs, in both examined tissue. Treatments with either with Cs or MIX Ld/Cs manage to retain the level of RAGE in both examined tissues at the control level (**Figure 6A**). Since RAGE activation perpetuates NF-κB activation, we examined the translocation of NF-κB p65 from the cytosol to the nucleus (**Figure 6B**). Western blot analysis revealed an overall increase in NF-κB p65 in liver and kidney nuclear fractions of diabetic rats (observed as an approximate nuclear:cytoplasmic p65 ratio >1), as compared to non-diabetic controls (where the nuclear:cytoplasmic p65 ratio < 1). While the cellular distribution of p65 changed, the amount of total p65 was not significantly altered in the livers of diabetic rats. However, in the kidney of diabetic rats, the amount of total p65 was higher than in control kidney. Administration of the extracts to diabetic rats produced varied responses. In the liver, their administration lowered the amount of total p65 significantly below the control level, with Cs producing the most pronounced decrease. Also, administration of extracts led to significant decrease of p65 in the liver nuclear fraction of diabetic rats, In the kidney, Cs administration in diabetic animal effectively lowered the relative amount of total p65 to the control level, bringing the nuclear:cytoplasmic p65 ratio <1. While Ld administration was less effective in lowering the relative amount of total p65, it also shifted the nuclear:cytoplasmic p65 ratio <1. The administration of MIX Ld/Cs had no effect in lowering the relative amount of total p65, but it shifted the nuclear:cytoplasmic p65 ratio <1.

**FIGURE 7:**
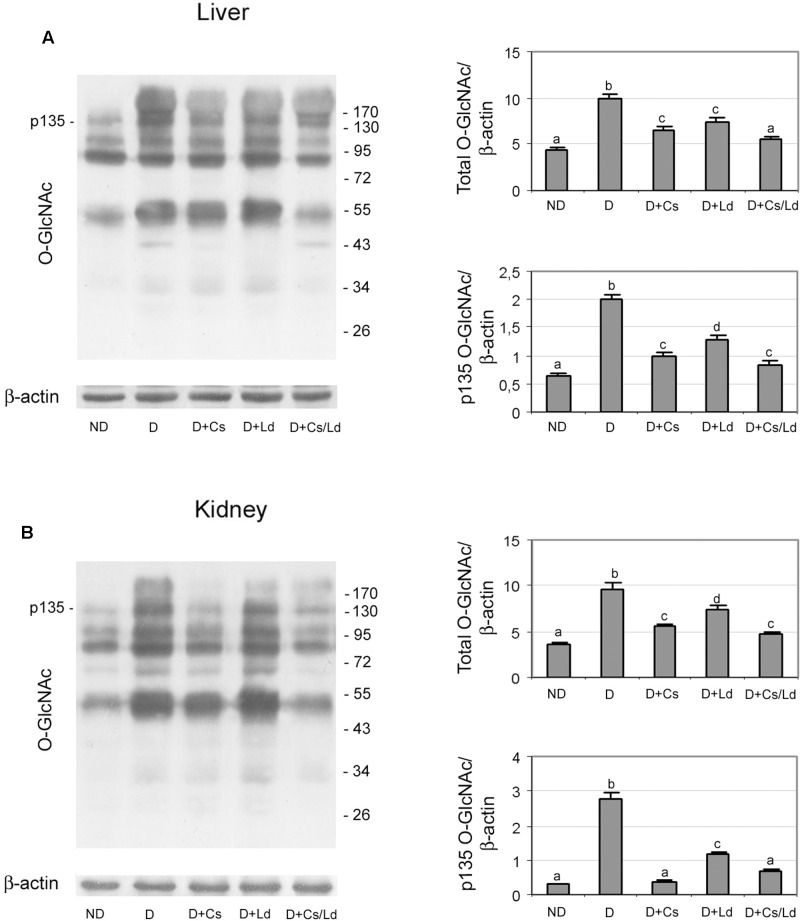
The effect of *C. sativa, L. deterrimus* extracts and their combination on the level of *O*-GlcNAc-modified proteins in liver **(A)** and kidney **(B)** tissue. The abundance of *O*-GlcNAc modified proteins in whole liver and kidney homogenates was assessed by immunoblot analysis. ND, non-diabetic control rats; D, diabetic rats; D + Cs, diabetic rats treated with *C. sativa*; D + Ld, diabetic rats treated with *L. deterrimus*; D + Cs/Ld, diabetic rats treated with the combination of *C. sativa* and *L. deterrimus* extracts. Results are expressed as means ± SEM. Means not sharing a common letter are significantly different between groups (DMRT; *p* < 0.05).

In addition to the AGE/RAGE pathway, we examined weather changes in AGE formation influenced enzymatic protein *O*-GlcNAcylation. To that end, we performed immunoblot analysis of liver and kidney homogenates with anti-*O*-GlcNAc antibody. Representative immunoblots for liver and kidney tissue are shown in **Figures 7A,B**, respectively. Densitometric analysis showed an increased level of total protein *O*-glycosylation in both tissue of diabetic rats (**Figures 7A,B**). The treatments of diabetic rats with the extracts significantly reduced the amount of protein *O*-glycosylation in both organs. The greatest reduction of *O*-GlcNAc proteins was detected in diabetic rats treated with the combination of mushroom and chestnut extracts. The observed changes in the level of *O*-GlcNAc proteins were most apparent in the band indicated as p135, which has been identified as *O-N*-acetylglucosamine transferase (Konrad et al., 2001; Zraika et al., 2006). Densitometric analysis of the protein band at 135 kDa showed a similar pattern of change as total *O*-GlcNAc proteins. However, the reduction of p135 glycosylation in both the liver and kidney of diabetic rats treated with Ld the extracts was more pronounced than the reduction of total *O*-GlcNAc proteins with regard to their levels in untreated, diabetic rats.

## Discussion

Chronic hyperglycemia, together with increased plasma cholesterol and triglyceride concentrations in STZ-induced diabetic rats, causes the generation of ROS which leads to cumulative oxidative damage of tissues and organs, producing long-term diabetic complications considered to be the major causes of morbidity and mortality in human diabetic populations (Lyra et al., 2006). In the present study, AST and ALT were used as marker enzymes to assess the extent of liver damage (Kim et al., 2006), while BUN served as a kidney injury biomarker (Rouse et al., 2013). The observed increased plasma levels of liver enzymes together with histological changes in liver tissue of diabetic rats revealed mild hepatocellular injury and changes in liver function (Kim et al., 2006), while the elevation of BUN together with observed histological alterations in kidney tissue points to renal dysfunction (Rouse et al., 2013). Hepatorenal injury was caused by oxidative damage, revealed by the elevated level of DCFDA estimated ROS production in diabetic rats which lead to increased lipid peroxidation and DNA damage in liver and kidney cells (Kakkar et al., 1995; Hulbert et al., 2007). The significant decreases in antioxidant enzyme activities and of the GSH/GSSG ratio suggest that oxidative stress-induced perturbations of liver and kidney functions were the result of the inability of the antioxidant defense systems to maintain redox balance.

The significantly lowered diabetic hyperglycemia and alleviated dyslipidemia after administration of the Cs and Ls extracts, either separately or together (MIX Ld/Cs), could be attributed to the systemic antioxidant effects of the extracts and their impact on pancreatic islets in diabetic rats. Our previous immunohistological examination of the pancreas showed that Ld administration to diabetic rats decreased islet destruction and partially restored the number of insulin-positive cells (Mihailovic et al., 2015), leading to better glycemic control, manifesting as an improvement of GlyHb and the lipid status, particularly after the Cs treatment. One reason for the lower effectiveness of the combination of extracts (MIX Cs/Ld) as compared to Cs alone in restoring glucose homeostasis could be due to the specific proportion of the two extracts in the mixture (50% each). Different ratios of Cs and Ld extracts could provide distinct responses (Vieira et al., 2012), and the lack of synergistic or additive effect may be due to the chemical nature and reactivity of compounds present in the mixtures (Nedamani et al., 2014). These compounds can suffer polymerization and other reactions causing structural alterations that result in changed antioxidant activities (Pinelo et al., 2004). On the other hand, treatment of diabetic rats with MixCs/Ld improved all three markers of hepatorenal function (AST, ALT, and BUN), Cs treatment improved two (ALT and BUN) and Ld treatment one (AST).

The improvement of hepatorenal function and structure in extract-treated diabetic rats was associated with recovery of the antioxidant defense systems. This was observed as, enhanced levels of antioxidant enzymes MnSOD and CuZnSOD, an improved GSH/GSSG ratio, and decreased oxidative damage of lipids and DNA, with the MIX Ld/Cs providing the best protection. The effect on GSH, the first line of defense against ROS, shifted the cellular redox balance of glutathione completely in favor of its reduced form in both tissues. GSH is also a cofactor of several detoxifying enzymes that provide the second line of antioxidant defense by detoxifying harmful byproducts generated by ROS and preventing the propagation of free radicals (Masella et al., 2005). The overall improvement of the antioxidant defense systems can also be attributed to the direct antioxidant effects of the extracts. Our previous work showed that the powerful antioxidant effect of the Cs extract was linked to the high content of phenolics and flavonoids (Grdović et al., 2012), and for the Ld extract, it was assumed that the enrichment in essential trace elements, such as Se and Zn, could have been responsible for the described beneficial antioxidant effect of the mushroom extract (Grdović et al., 2012). Further, edible mushrooms contain an abundance of functional components such as β-glucans (Manzi and Pizzoferrato, 2000) that play an important role in the recovery of the antioxidant defense system in the liver and kidney of diabetic rats (Mihailovic et al., 2013a).

Phenolic metabolites with antioxidant activities also mediate the antiglycation activity of medicinal plants (Harris et al., 2011). The phenolic-rich Cs extract (Grdović et al., 2012) decreased most efficiently the formation of fructosamines and total AGE in an *in vitro* glycation process, which is consonant with its ability to maintain fasting blood glucose at the control level in diabetic rats, and to prevent AGE formation in blood where glycation of long-lived proteins such as Hb contributes to vascular damage and the development of diabetic complications. The antiglycation properties of the examined extracts present a good therapeutic potential in the treatment of diabetes (Ramasamy, 2006; Mihailovic et al., 2013b).

Oxidation, which accompanies glycation *in vivo*, supports the formation of more permanent, irreversible chemical modifications, such as the well-characterized CML that serves as a marker of AGE (Chappey et al., 1997). We detected significantly increased levels of CML in liver and kidney tissues of diabetic rats. Because the liver has a pivotal role in AGE catabolism, the consequence of impaired hepatic function could be an increase in the levels of circulating AGEs which leads to their accumulation in other tissues. Almost all renal structures are susceptible to accumulate AGEs, particularly CML under diabetic conditions (Horie et al., 1997).

Aside from established direct toxicity, certain AGE, such as CML-modified proteins, are powerful inducers of RAGE signaling (Herold et al., 2007). One of the consequences of AGE–RAGE interaction is the generation of ROS (Yan and Harding, 1997; Jiang et al., 2004). AGE also perpetuate NF-κB activation by *de novo* synthesis of NF-κB p65 (Bierhaus et al., 2001), which promotes the expression of NF-κB-regulated genes, including RAGE itself. Overexpression of RAGE enhances the cell’s capacity to bind AGE, and initiates a vicious cycles that perpetuates oxidative stress leading to tissue and organ dysfunction in diabetes. In studies of diabetes-associated renal diseases, the upregulation of RAGE has been linked to enhanced levels of AGE (Jerums et al., 2003) and pronounced tissue injury, which could be reduced by a functional RAGE blockade (Kuhla et al., 2010). Our results show that treatment with either extract interrupts the vicious cycle of CML-mediated RAGE/NF-κB activation by decreasing protein glycation and CML formation in both tissues, indicating that both examined extracts express strong antiglycating activity *in vivo*. Reduction of CML levels, particularly as observed in Cs extract-treated diabetic rats, could be the outcome of improved hyperglycemia. On the other hand, the prominent antiglycating activity of the Ld extract that was observed in liver and kidney tissues of diabetic rats, in spite of its weaker antihyperglycemic activity, could be the result of its antioxidant effect and improved redox status.

The cell senses concentration changes of excess glucose under hyperglycemic conditions via elevated levels of uridine diphosphate-β-*N*-acetylglucosamine (UDP-GlcNAc), which is the substrate for *O*-GlcNAc transferase (OGT) that catalyzes the formation of a post-translational *N*-acetyl-glucosamine modification of serine and threonine residues of nuclear and cytoplasmic proteins. The increase in *O*-GlcNAc-modified proteins in the liver and kidney of diabetic rats is probably the result of increased activity of OGT (Kreppel and Hart, 1999; Konrad et al., 2001), while the significant decrease in *O*-glycosylation in liver and kidney tissue of diabetic rats after extract application could be the result of decreased *O*-GlcNAcylation of OGT. Because glycosylation alters the behavior of specific proteins by modulating their activities, protein–protein interactions, DNA-binding activity, subcellular localization and half-lives and proteolytic processing (Zachara and Hart, 2004; Li et al., 2007), by limiting the level of *O*-GlcNAcylation, the extracts play an important role in maintaining normal cell signaling pathways under diabetic conditions, with the MIX Ld/Cs providing the best inhibitory effect on *O*-GlcNAcylation.

The presented data show that the administration of chestnut and mushroom extracts, either individually or together, improved the diabetes-induced pathological profile of liver and kidney cells. Generally, treatment of diabetic rats with Cs extract compared to the Ld extract provided better protection and treatment with Mix Ld/Cs was equal or better that Cs. The beneficial effect of the examined extracts was achieved not only by reducing the glucose-triggered overproduction of free radicals and enhancing the effectiveness of the cell’s antioxidant defenses, but also through a marked antiglycation activity. Also, the extracts significantly reduced non-enzymatic glycosylation in diabetic rats *in vivo*, which leads to inhibition of CML-mediated RAGE/NF-κB activation and enzymatic *O*-GlcNAcylation in liver and kidney tissues. This should have positive impact on various detrimental cellular processes, delaying the progression of the diabetic complications. These findings may be relevant with regard to the therapeutic potential of chestnut and mushroom extracts in treating the diabetic condition and also in the management of oxidative stress in other pathological states.

## Author Contributions

JJ, MV, and MM planned the study and designed the experiments. IM collected the samples for the extracts and performed the HPLC analysis. JJ and MM carried out the biochemical blood analysis, histological analysis, estimation of oxidative stress and antioxidant enzyme activities and glycation-related *in vitro* analysis. NG, SD, and AU determined the level of oxidative damage of lipids and DNA and GSH/GSSG ratio and performed the immunoblot analysis. JJ, MV, and GP summarized, discussed and interpreted the results, and wrote the manuscript.

## Conflict of Interest Statement

The authors declare that the research was conducted in the absence of any commercial or financial relationships that could be construed as a potential conflict of interest.

## Footnotes

1 http://www.AutoComet.com
